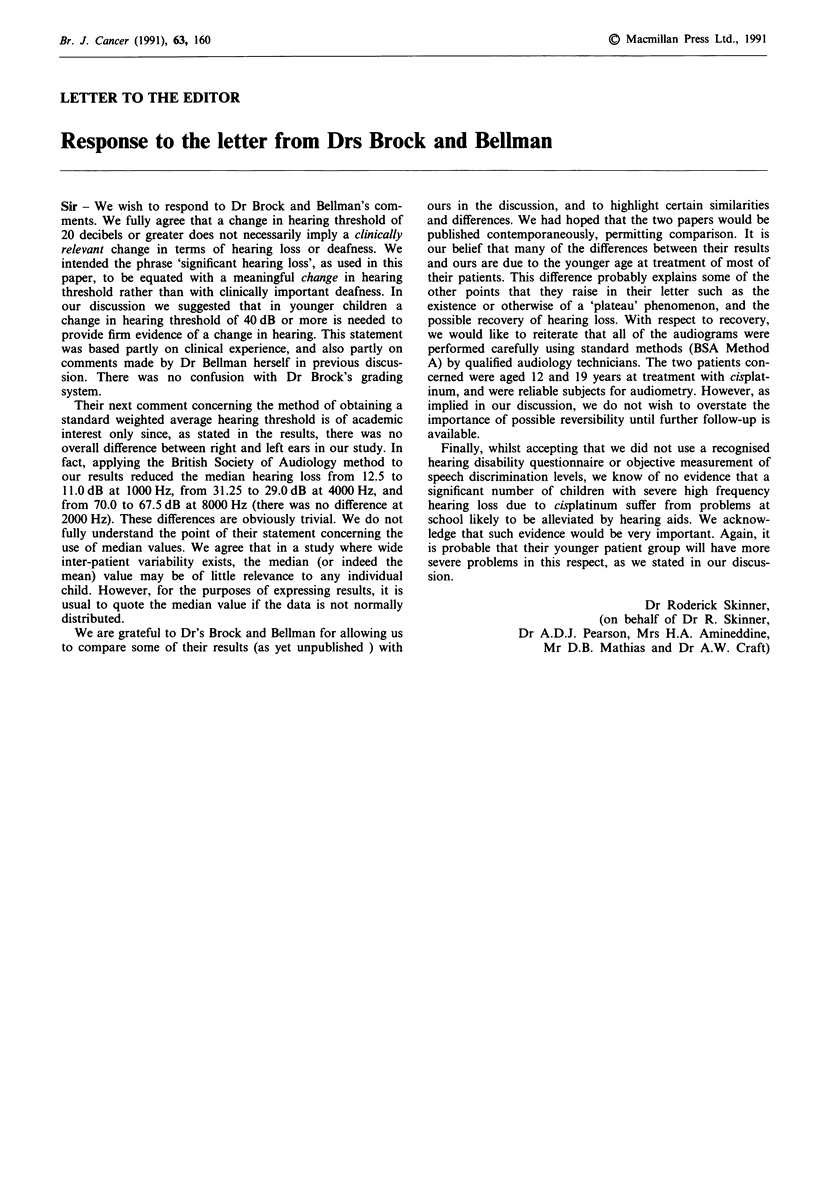# Response to the letter from Drs Brock and Bellman

**Published:** 1991-01

**Authors:** Roderick Skinner


Br. J. Cancer (1991), 63, 160

© Macmillan Press Ltd., 1991

LETTER TO THE EDITOR

### Response to the letter from Drs Brock and Bellman

**Sir** – We wish to respond to Dr Brock and Bellman's comments. We fully agree that a change in hearing threshold of 20 decibels or greater does not necessarily imply a *clinically relevant* change in terms of hearing loss or deafness. We intended the phrase 'significant hearing loss', as used in this paper, to be equated with a meaningful *change* in hearing threshold rather than with clinically important deafness. In our discussion we suggested that in younger children a change in hearing threshold of 40 dB or more is needed to provide firm evidence of a change in hearing. This statement was based partly on clinical experience, and also partly on comments made by Dr Bellman herself in previous discussion. There was no confusion with Dr Brock's grading system.

Their next comment concerning the method of obtaining a standard weighted average hearing threshold is of academic interest only since, as stated in the results, there was no overall difference between right and left ears in our study. In fact, applying the British Society of Audiology method to our results reduced the median hearing loss from 12.5 to 11.0 dB at 1000 Hz, from 31.25 to 29.0 dB at 4000 Hz, and from 70.0 to 67.5 dB at 8000 Hz (there was no difference at 2000 Hz). These differences are obviously trivial. We do not fully understand the point of their statement concerning the use of median values. We agree that in a study where wide inter-patient variability exists, the median (or indeed the mean) value may be of little relevance to any individual child. However, for the purposes of expressing results, it is usual to quote the median value if the data is not normally distributed.

We are grateful to Dr's Brock and Bellman for allowing us to compare some of their results (as yet unpublished ) with ours in the discussion, and to highlight certain similarities and differences. We had hoped that the two papers would be published contemporaneously, permitting comparison. It is our belief that many of the differences between their results and ours are due to the younger age at treatment of most of their patients. This difference probably explains some of the other points that they raise in their letter such as the existence or otherwise of a 'plateau' phenomenon, and the possible recovery of hearing loss. With respect to recovery, we would like to reiterate that all of the audiograms were performed carefully using standard methods (BSA Method A) by qualified audiology technicians. The two patients concerned were aged 12 and 19 years at treatment with *cis*platinum, and were reliable subjects for audiometry. However, as implied in our discussion, we do not wish to overstate the importance of possible reversibility until further follow-up is available.

Finally, whilst accepting that we did not use a recognised hearing disability questionnaire or objective measurement of speech discrimination levels, we know of no evidence that a significant number of children with severe high frequency hearing loss due to *cis*platinum suffer from problems at school likely to be alleviated by hearing aids. We acknowledge that such evidence would be very important. Again, it is probable that their younger patient group will have more severe problems in this respect, as we stated in our discussion.

Dr Roderick Skinner,
(on behalf of Dr R. Skinner,
Dr A.D.J. Pearson, Mrs H.A. Amineddine,
Mr D.B. Mathias and Dr A.W. Craft)